# Circulating lncRNA UCA1 and lncRNA PGM5-AS1 act as potential diagnostic biomarkers for early-stage colorectal cancer

**DOI:** 10.1042/BSR20211115

**Published:** 2021-07-12

**Authors:** Minghui Wang, Zhijun Zhang, Deng Pan, Zhigang Xin, Fan Bu, Yue Zhang, Qingwu Tian, Xiaodong Feng

**Affiliations:** 1 Department of Clinical Laboratory, The Affiliated Hospital of Qingdao University, Qingdao University, Qingdao 266000, China; 2 Department of Clinical Laboratory, Taian City Central Hospital, Taian 271000, China

**Keywords:** biomarkers, CRC, lncRNA, ROC curve

## Abstract

**Background:** Colorectal cancer (CRC) is one of the most common and significant malignant diseases worldwide. In the present study, we evaluated two long non-coding RNAs (lncRNAs) in CRC patients as diagnostic markers for early-stage CRC.

**Methods:** Using Gene Expression Omnibus (GEO) datasets GSE102340, GSE126092, GSE109454 and GSE115856, 14 differentially expressed lncRNAs were identified between cancer and adjacent tissues, among which, the two most differentially expressed were confirmed using quantitative real-time polymerase chain reaction (qRT-PCR) in 200 healthy controls and 188 CRC patients. A receiver operating characteristic (ROC) analysis was employed to evaluate the diagnostic accuracy for CRC.

**Results:** From four GEO datasets, three up-regulated and eleven down-regulated lncRNAs were identified in CRC tissues, among which, lncRNA urothelial carcinoma-associated 1 (UCA1) and lncRNA phosphoglucomutase 5-antisense RNA 1 (PGM5-AS1) were the most significantly up- and down-regulated lncRNAs in CRC patient plasma, respectively. The area under the ROC curve was calculated to be 0.766, 0.754 and 0.798 for UCA1, PGM5-AS1 and the combination of these two lncRNAs, respectively. Moreover, the diagnostic potential of these two lncRNAs was even higher for the early stages of CRC. The combination of UCA1 and PGM5-AS1 enhanced the AUC to 0.832, and when the lncRNAs were used with carcinoembryonic antigen (CEA), the AUC was further improved to 0.874.

**Conclusion:** In the present study, we identified two lncRNAs, UCA1 and PGM5-AS1, in CRC patients’ plasma, which have the potential to be used as diagnostic biomarkers of CRC.

## Introduction

As one of the most deadly cancer, roughly 1.8 million new cases of colorectal cancer (CRC) have been diagnosed, and more than 900000 patients died because of CRC in 2020 [1]. Although many effective new treatments have been developed in recent decades, the long-term survival rate is still low due to the lack of effective early-stage diagnosis methods [2]. Body fluid-based testing is an inexpensive and non-invasive method for the early-stage diagnosis of cancer and provides crucial information for tumor process monitoring and prognostic evaluation [3]. For example, the detection of α-fetoprotein (AFP) is of great importance in the early-stage diagnosis of hepatocellular carcinoma [4]. However, the currently used serum tumor biomarkers, carbohydrate antigen 19-9 and carcinoembryonic antigen (CEA), have shown a low positive rate and poor sensitivity toward the early-stage CRC. Hence, there is an urgent need to find new biomarkers with high sensitivity and specificity for the early-stage diagnosis of CRC.

Long non-coding RNAs (lncRNAs) [5,6] play an important role in the regulation of gene expression through chromatin modification [7–9]. Recent studies have revealed that lncRNAs can regulate multiple biological pathways, such as metabolism processes, cellular growth, differentiation and apoptosis, and play important regulatory roles in cancer [10–14]. Circulating lncRNAs have been used as cancer markers for the early-stage diagnosis of different forms of cancer, such as CRC and breast cancer [15–18].

In the present study, we aimed to find novel plasma lncRNAs as biomarkers for the early-stage diagnosis of CRC. By analyzing four Gene Expression Omnibus (GEO) datasets, we obtained a number of differentially expressed lncRNAs, among which lncRNA urothelial carcinoma-associated 1 (UCA1) and phosphoglucomutase 5-antisense RNA 1 (PGM5-AS1) were confirmed using quantitative real-time polymerase chain reaction (qRT-PCR). These lncRNAs show great potentials as diagnostic biomarkers for the early-stage CRC and may provide new insights into the pathological mechanism of CRC.

## Materials and methods

### LncRNAs expression in public datasets

The expression profiles matching ‘colorectal cancer’ and ‘LncRNA’ in the GEO public dataset were used to select and analyze differentially expressed genes in CRC tissues and adjacent tissues, by which, four datasets, GSE102340, GSE126092, GSE109454 and GSE115856, were selected. The information of the four GEO datasets is summarized in Supplementary Table S1. GEO2R was employed to analyze differentially expressed genes among each dataset using the following criteria: logFC ≥ 1, *P*-value <0.05 for up-regulated genes and logFC ≤ −1, *P*-value <0.05 for down-regulated genes. Then, the online Venn diagram tool (http://bioinformatics.psb.ugent.be/webtools/Venn/) was used to identify the common differentially expressed genes from these four datasets.

### Sample collection

The present study was conducted on 188 CRC patients who were enrolled in the Affiliated Hospital of Qingdao University from 3 April 2019 to 30 September 2020. The CRC patients were diagnosed by two independent pathologists. A total of 200 healthy individuals who participated in health examination in the Health Management Center were selected as the control. The qualified controls should not have any related diseases such as immunodeficiency diseases, hypertension and diabetes. All the CRC patients were not treated before sampling.

### RNA isolation

Plasma was collected from CRC patients and controls in separating gel coagulation-promoting tubes, which was used for Total RNA preparation using a TIANGEN RNA extraction kit (Beijing, China). A NanoDrop OneC Spectrophotometer (Thermo Scientific, Rockford, IL) was used to measure the quantity and purity of the RNA. The OD260/OD280 ratio of the samples used for reverse transcription was between 1.8 and 2.0.

### qRT-PCR

The complementary DNA (cDNA) for real-time PCR was generated from 1.0 μg RNA sample using the PrimeScript RT reagent Kit with gDNA Eraser (#RR047A, Takara, Tokyo, Japan) which is a reverse-transcription kit for real-time RT-PCR (RT-qPCR) that includes a genomic DNA elimination reaction. The real-time PCR was performed starting with an initial denaturation step at 95°C for 30 s, followed by 45 cycles at 95°C for 5 s, and 60°C for 30 s. Glyceraldehyde-3-phosphate dehydrogenase (GAPDH) was used as the internal control. LncRNAs with non-specific amplification and primer dimerization were not included in the present study. In addition, lncRNAs that were hardly detected by qRT-PCR due to their low copy number (*C*_t_ value > 35) were excluded from the present study. The primers of the lncRNAs were designed on exon regions spanning introns to avoid amplification derived from genomic DNA. The primers are as follows: UCA1, forward: 5ʹ- GCCGAGAGCCGATCAGACAAAC-3ʹ, reverse: 5ʹ-AACGGATGAAGCCTGCTTGGAAG-3ʹ; PGM5-AS1, forward: 5ʹ-AGCTGGTGGAATCATTCTAACAʹ, reverse: 5ʹ-GAGATAGGTCGATTCGGAGATCʹ; GAPDH, forward: 5ʹ-TGACTTCAACAGCGACACCCA-3ʹ, reverse: 5ʹ-CACCCTGTTGCTGTAGCCAAA-3ʹ. The relative lncRNA levels were calculated by the 2^−ΔΔ*C*_t_^ method.

### Statistical analysis

The qRT-PCR results of the CRC patients were compared with those of the healthy individuals by the Mann–Whitney U test. The diagnostic potentials of the lncRNAs were evaluated using the ROC curves. The experimental results were presented as the mean ± standard deviation (SD), and the *P*<0.05 was considered to be statistically significant. The statistical analyses were performed using the SPSS package, version 19.0 (Chicago, IL).

## Results

### Screened out differentially expressed lncRNAs

**Figure 1:**
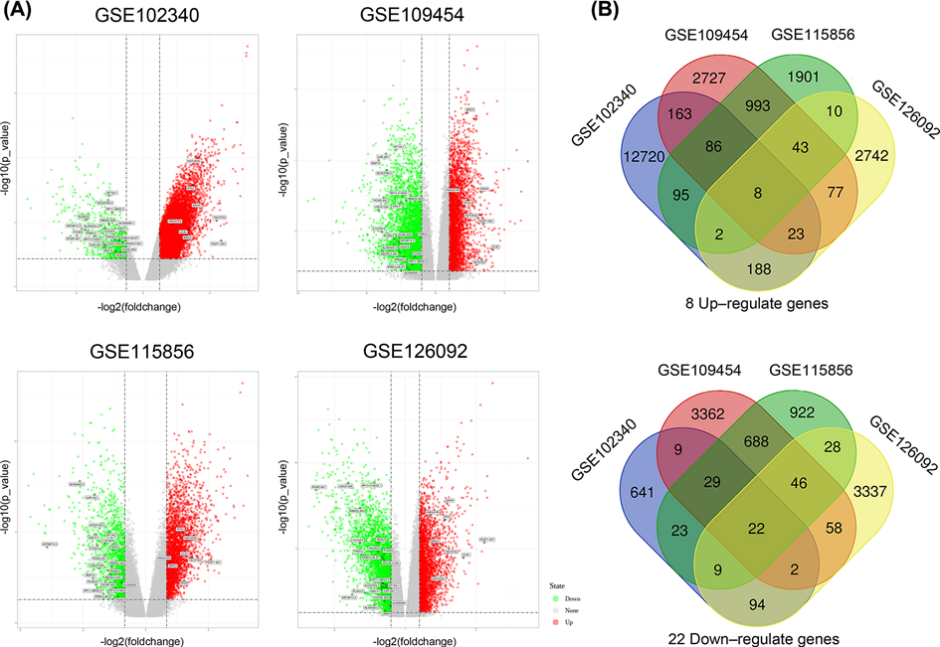
Differentially expressed genes in the four datasets and the result of their intersection (**A**) Volcano plots of the differentially expressed genes for GSE102340, GSE126092, GSE109454, and GSE115856. (**B**) Venn diagrams for down- and up-regulated genes.

The differentially expressed genes from each dataset were analyzed using the NCBI online tool GEO2R, and the expression profiles are presented as volcano plots as shown in Figure 1A. The Venn diagrams for the up- and down-regulated genes were plotted, from which, 8 genes were obtained by intersecting the up-regulated genes in the four datasets, and 22 genes were obtained by intersecting the down-regulated genes (Figure 1B). A total of 14 lncRNAs were assigned as common differentially expressed genes, inluding 3 up-regulated lncRNAs, UCA1, HMGA1P4 and FEZF1-AS1 and 11 down-regulated lncRNAs, RP11-38P22.2, RP13-514E23.1, PGM5-AS1, HOXD-AS2, RBMS3-AS3, IL6R-AS1, LINC01354, AC023794.2, AL050403.2, EPB41L4A-DT, and ADGRL3-AS1 (Table 1).

**Table 1:** LncRNAs expression in four datasets

LncRNAs	GSE102340			GSE109454			GSE115856			GSE126092		
	logFC	Description	*P*-value	logFC	Description	*P*-value	logFC	Description	*P*-value	logFC	Description	*P*-value
UCA1	4.68	Up	7.17E-03	3.47	Up	5.13E-04	3.11	Up	3.06E-04	5.58	Up	4.24E-06
HMGA1P4	1.74	Up	1.24E-03	1.05	Up	2.53E-05	1.01	Up	1.72E-04	1.74	Up	1.10E-07
FEZF1-AS1	2.75	Up	2.09E-03	4.15	Up	6.29E-03	1.99	Up	2.20E-04	4.09	Up	3.26E-05
RP11-38P22.2	−1.85	Down	3.35E-04	−1.69	Down	2.66E-02	−2.31	Down	1.10E-02	−1.62	Down	9.67E-04
RP13-514E23.1	−2.37	Down	8.69E-03	−2.38	Down	3.36E-05	−1.44	Down	1.07E-02	−2.18	Down	1.37E-09
PGM5-AS1	−3.97	Down	4.11E-03	−3.77	Down	3.82E-05	−1.98	Down	1.82E-04	−6.29	Down	3.86E-09
HOXD-AS2	−1.38	Down	2.41E-02	−2.61	Down	1.92E-03	−1.91	Down	5.23E-03	−1.45	Down	1.02E-02
RBMS3-AS3	−2.03	Down	1.31E-03	−2.15	Down	5.29E-05	−1.61	Down	7.17E-04	−3.19	Down	4.09E-08
IL6R-AS1	−2.28	Down	1.15E-02	−3.59	Down	5.32E-07	−2.44	Down	8.32E-08	−1.35	Down	1.51E-03
LINC01354	−2.19	Down	1.16E-02	−4.13	Down	1.29E-04	−2.21	Down	1.00E-03	−4.03	Down	1.53E-09
AC023794.2	−2.10	Down	9.14E-05	−3.52	Down	2.65E-06	−2.20	Down	6.06E-06	−1.49	Down	1.73E-03
AL050403.2	−2.51	down	1.41E-03	−3.04	Down	3.81E-03	−3.15	Down	1.54E-08	−2.17	Down	1.73E-02
EPB41L4A-DT	−1.44	down	7.28E-03	−2.06	Down	3.52E-02	−2.02	Down	2.32E-02	−1.60	Down	2.66E-05
ADGRL3-AS1	−1.23	down	8.65E-03	−2.61	Down	7.51E-03	−1.69	Down	2.02E-03	−1.57	Down	3.71E-05

### Validation of the diagnostic potential of the lncRNAs

**Figure 2:**
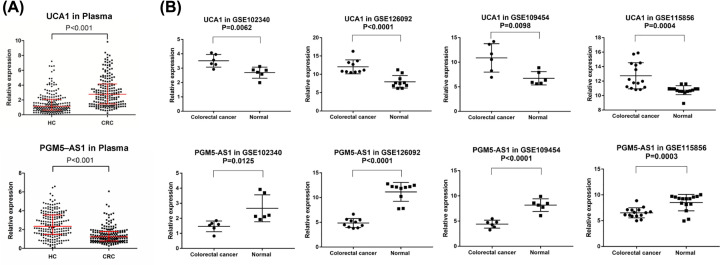
Relative expression of UCA1 and PGM5-AS1 in plasma and in the four datasets (**A**) The expression levels of UCA1 and PGM5-AS1 in 188 CRC patients and 200 healthy donors. (**B**) The relative expression of UCA1 and PGM5-AS1 in GSE102340, GSE126092, GSE109454, and GSE115856 datasets.

To validate the potential of these lncRNAs in the diagnosis of CRC, we measured their expression level in the plasma of 188 CRC patients and 200 healthy donors. Among these lncRNAs, the expression of UCA1 and PGM5-AS1 was greatly up- and down-regulated, respectively (*P*<0.001, Figure 2A). We also analyzed their expression in four GEO datasets, which confirmed the elevated expression of UCA1 in CRC tumor and the decreased expression of PGM5-AS1 in GSE102340, GSE126092, GSE109454, and GSE115856 datasets (Figure 2B).

### Correlation between UCA1 and PGM5-AS1 expression and clinicopathological characteristics

The correlation between the expression levels of UCA1 and PGM5-AS1 and the clinicopathological characteristics of CRC patients are analyzed (Table 2), which showed that the expression of UCA1 nor PGM5-AS1 had no correlation with age, gender, differentiation, and AJCC stage of the patients.

**Table 2:** Relationship between clinicopathological characteristics and UCA1 and PGM5-AS1 expression (*n*=188)

Variable	Cases	UCA1	*P-*value*	PGM5-AS1	*P*-value*
		Median with interquartile range		Median with interquartile range	
Age					
<60	65	2.789 (1.474–4.634)	0.279	1.275 (0.785–1.840)	0.684
≥60	123	3.639 (1.434–4.170)		1.223 (0.780–1.778)	
Gender					
Male	106	2.594 (1.331–4.042)	0.441	1.117 (0.731–1.778)	0.662
Female	82	3.010 (1.566–4.580)		1.324 (0.989–1.850)	
Differentiation					
Well	87	2.338 (1.425–3.925)	0.208	1.218 (0.773–1.759)	0.898
Moderate+Poor	101	2.819 (1.464–4.619)		1.253 (0.803–1.840)	
AJCC stage					
I + II	128	2.639 (1.459–4.077)	0.904	1.137 (0.776–1.600)	0.064
III + IV	60	2.858 (1.444–4.402)		1.532 (0.844–2.014)	

*P-values are based on unpaired samples *t* test.

### Diagnostic potential of UCA1 and PGM5-AS1

**Figure 3:**
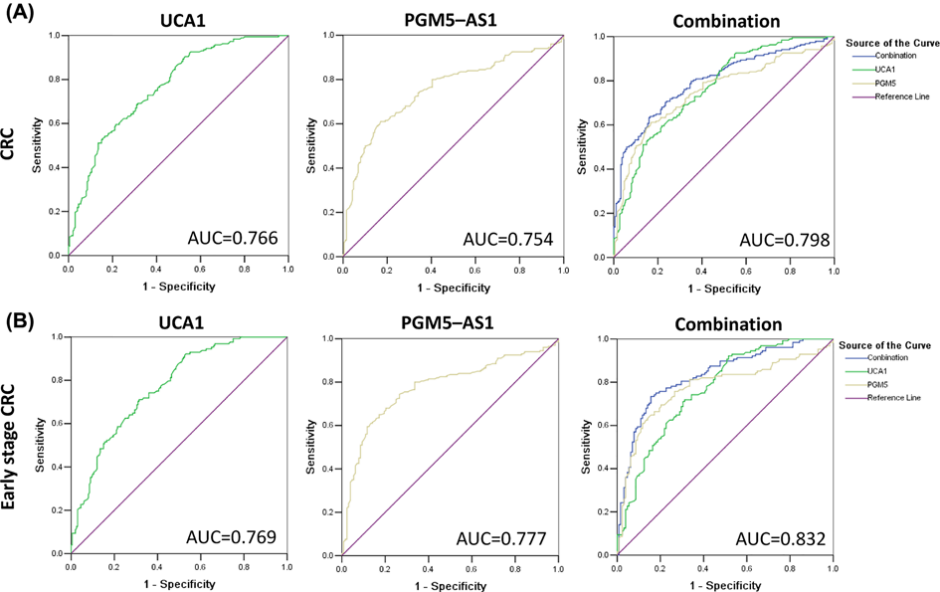
Diagnostic value of the UCA1 and PGM5-AS1 in plasma for CRC (**A**) AUC for UCA1, PGM5-AS1 and the combination of UCA1 and PGM5-AS1 for CRC. (**B**) AUC for UCA1, PGM5-AS1 and the combination of UCA1 and PGM5-AS1 for early-stage CRC (stages I and II).

The diagnostic potential of the differentially expressed lncRNAs was assessed using ROC curve analysis, which showed that the area under the ROC curve (AUC) was 0.766 (95% CI: 0.712–0.813) and 0.754 (95% CI: 0.705–0.803) for UCA1 and PGM5-AS1, respectively. The AUC value could be improved to 0.798 (95% CI: 0.753–0.843) by combining the values of UCA1 and PGM5-AS1 (Figure 3A). In addition, the diagnostic potential for the early stage of CRC was measured, which showed that AUCs were 0.769 (95% CI: 0.712–0.826) and 0.777 (95% CI: 0.717–0.836) for UCA1 and PGM5-AS1, respectively. Similarly, by the combination of UCA1 and PGM5-AS1, the AUC valued could be improved to 0.832 (95% CI: 0.782–0.882) for the early stage (stage I and II) CRC diagnosis (Figure 3B).

### Diagnostic value of lncRNAs with conventional biomarkers

**Figure 4:**
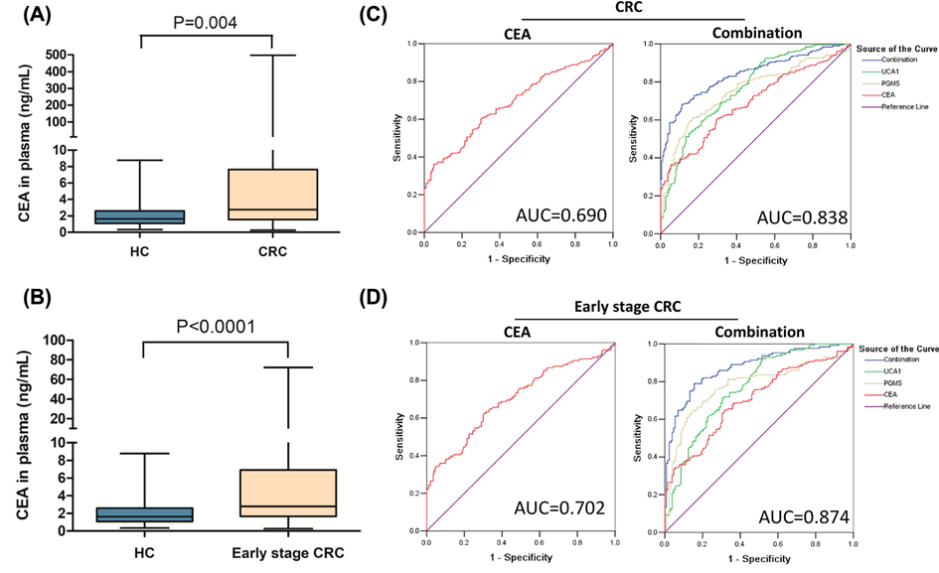
Diagnostic value of the combination of UCA1, PGM5-AS1 and serum CEA for CRC (**A**) Expression of CEA in the plasma of CRC patients. (**B**) Expression of CEA in the plasma of early-stage CRC patients. (**C**) Diagnostic value of the combination of UCA1, PGM5-AS1 and CEA for CRC. (**D**) Diagnostic value of the combination of UCA1, PGM5-AS1 and CEA for early-stage CRC (stages I and II).

CEA is one of the most commonly used CRC markers in clinical applications. Thus, we assessed its diagnostic value in combination with UCA1 and PGM5-AS1. The results indicated that the CEA expression in plasma in the healthy controls was significantly lower than that of the CRC patients as well as the early-stage patients (Figure 4A,B). The AUC of CEA alone was 0.690 (95% CI: 0.636–0.743), which could be greatly increased to 0.838 (95% CI: 0.798–0.879) (Figure 4C) when used in combination with UCA1 and PGM5-AS1. In addition, by using the combination with UCA1 and PGM5-AS1, the diagnostic value of the early-stage CRC could be improved to 0.874 (95% CI: 0.831–0.917) (Figure 4D).

## Discussion

Molecular diagnostic markers have been developed for different forms of tumors, and some of them have been used clinically [19]. However, most of these biomarkers are not adequately sensitive for the diagnosis of early-stage tumors [20]. Thus, new and more sensitive markers are urgently needed [21]. LncRNAs have shown great potential in the diagnosis of various tumors, such as bladder cancer, lung cancer and prostate cancer [16,17,22–27]. In this study, we analyzed a total of 74 samples from GSE102340, GSE126092, GSE109454, and GSE115856 datasets to find potential lncRNA markers for the diagnosis of CRC. We identified two lncRNAs with the most significant changes of expression, UCA1 and PGM5-AS1, which could be used as potential markers for the diagnosis of early-stage CRC.

UCA1 belongs to the human endogenous retrovirus H family and is localized on chromosome 19p13.12 [28]. Studies have shown that that UCA1 was universally expressed in embryonic tissues [29]. Recently, accumulating reports have revealed that UCA1 may function as an oncogenic lncRNA in the occurrence and progression of tumors, as well as a prognostic marker as its expression is highly correlated with high metastatic propensity and poor survival rate of cancer patients at the advanced TNM stages [30–32]. Wang et al*.* reported that overexpression of UCA1 promotes migration and proliferation of gastric cancer cells, indicating UCA1 may be used as a therapeutic target for gastric cancer [33]. Xue et al*.* have found that the expression of UCA1 is elevated in the hypoxic bladder cancer cell-derived exosomes compared with that of the healthy donors, which promotes bladder tumor growth though epithelial–mesenchymal transition (EMT) [34]. Our study showed that, compared with the healthy donors, the plasma expression of UCA1 was also increased in CRC patients, especially in those early-stage CRC patients (stages I and II).

Metabolic reprogramming has been regarded as an important hallmark of cancers [35], during which, phosphoglucomutase (PGM) plays a key role in glucose-1-phosphate and glucose-6-phosphate metabolism. LncRNA PGM5-AS1 has been identified as a tumor suppressor in CRC by a number of studies [36], as overexpression of PGM5-AS1 can inhibit CRC cells growth [37]. The expression of PGM5-AS1 may be positively correlated PGM5 expression, both of which are significantly down-regulated in CRC patients. It was also reported that PGM5-AS1 is down-regulated in CRC tissues and cells. PGM5-AS1 may promote tumor proliferation, migration and invasion by modulating the inhibitory effect of miR-100-5p on the tumor suppressor gene *SMAD4* [38]. These findings indirectly indicate that, consistent with our study, low expression of PGM5-AS1 is beneficial to CRC patients. However, the effects of PGM5-AS1 on CRC progression lie on multiple aspects. For example, Zhu et al*.* have demonstrated that PGM5-AS1 may be associated with CRC progression. Moreover, high PGM5-AS1 expression levels were associated with worse overall survival in CRC. It could be used as a novel potential therapeutic and prognostic target for CRC [39]. Therefore, the underlying molecular mechanism responsible for the different functions of PGM5-AS1 in CRC required needs further study.

It should be noted that there are some restrictions of our study. Firstly, clinical verification of the lncRNAs by using larger sample sizes is needed before they can be applied in clinical practice. Secondly, further work is required to identify more key lncRNAs related to the early-stage diagnosis of CRC and make the best combination to improve the diagnostic efficiency. Thirdly, the underlying mechanisms of differentially expressed lncRNAs involved in tumorigenesis are still not well understood and require further investigation.

## Conclusion

In summary, we identified two differentially expressed lncRNAs, UCA1 and PGM5-AS1, in the plasma of CRC patients, which showed great diagnostic potential of CRC, and by combining with traditional markers, the diagnosis of CRC, especially the early-stage CRC, could be improved.

## Supplementary Material

Supplementary Table S1

## Data Availability

All data analysed in the present paper are already included in the manuscript, including two tables.

## Abbreviations

AUC: area under curve
CEA: carcinoembryonic antigen
CRC: colorectal cancer
GAPDH: glyceraldehyde-3-phosphate dehydrogenase
GEO: Gene Expression Omnibus
lncRNA: long non-coding RNA
OD: optical density
PGM5-AS1: phosphoglucomutase 5-antisense RNA 1
qRT-PCR: quantitative real-time polymerase chain reaction
ROC: receiver operating characteristic
TNM: tumor node metastasis
UCA1: urothelial carcinoma-associated 1
