# Supplementary material for: Circulating lncRNA UCA1 and lncRNA PGM5-AS1 act as potential diagnostic biomarkers for early-stage colorectal cancer
Source: Biosci Rep. 2021 Jul 12;41(7):BSR20211115. doi: 10.1042/BSR20211115 (PMC8276091; doi:10.1042/BSR20211115)
Supplement: Supplementary Table S1 [file BSR-2021-1115_supp.pdf]

Table S1. Information of the four GEO datasets

Datasets	GSE102340	GSE109454	GSE115856	GSE126092
Published date	Aug 08, 2017	Jan 20, 2018	Jun 15, 2019	Feb 06, 2019
Platforms	GPL13825	GPL16956	GPL16956	GPL21047
Organism	Homo sapiens	Homo sapiens	Homo sapiens	Homo sapiens
Patients source	Guangzhou, China	Guangzhou, China	Guangzhou, China	Soochow, China
Samples	6 CRC tissues and matched normal tissues	6 CRC tissues and matched normal tissues	15 CRC tissues and matched normal tissues	10 CRC tissues and matched normal tissues
